# From milk to solid: a review and meta-regression analysis on the effects of solid feed on veal calf welfare and abomasal lesions

**DOI:** 10.3389/fvets.2025.1729514

**Published:** 2025-12-11

**Authors:** Giulio Cozzi, Luisa Magrin, Caterina Marcon, Barbara Contiero

**Affiliations:** Department of Animal Medicine, Production and Health, University of Padua, Legnaro (PD), Italy

**Keywords:** abomasal lesions, animal welfare, meta-regression analysis, solid feed, veal calves

## Abstract

This study reviewed the evolution of solid feed (SF) use in veal calf nutrition and conducted a regression meta-analysis to assess its effects on abomasal health. The traditional feeding system for veal calves consisted exclusively of liquid diets based on whole cow’s milk, and more recently on formulated milk replacers. The provision of limited quantities of SF (50–250 g per calf per day) was mandated by European Union welfare regulations with the aim of improving physiological and behavioral development. Data from 13 studies published between 2000 and 2024 were analyzed to quantify the relationship between SF supply and abomasal lesions. The dataset revealed that during this time period the mean daily SF intake increased by approximately 700 g per calf. The meta-regression analysis showed a strong positive correlation between the daily dry matter of SF administered and the prevalence of abomasal damage, indicating that each additional 100 g of SF dry matter corresponded to a 4 ± 1 percentage-point increase in lesion prevalence. Starch intake, rather than neutral detergent fiber (NDF) from SF, was significantly associated with lesion occurrence (*p* < 0.001). These findings suggest that while the inclusion of solids improved welfare compared with milk-only diets, current high-starch feeding practices compromise gastrointestinal integrity, highlighting the need to redefine optimal SF composition and inclusion levels for sustainable and welfare-oriented veal production.

## 1 Introduction

Livestock systems must achieve economic, environmental, and social sustainability (1). Animal welfare is central to this social dimension and is now embedded in public policy across many countries (2). The attitudes and expectations of citizens, consumers, veterinarians, farmers, and other stakeholders influence both livestock management practices and market dynamics for animal-derived foods, shaping willingness to pay and purchasing behavior (3). In the field of farm animal welfare, veal calves represent a relevant case study, as they are a livestock category for which housing systems and feeding practices have been modified within the European Union through specific regulatory directives. Based on the available scientific literature, this review article first analyses the evolution of solid feed (SF) administration in the diet of veal calves and the related positive and negative implications for animal health and welfare. In a second part, using a random-effects meta-regression analysis, the study aimed at quantifying the relationship between type and amount SF supply and abomasal damages.

## 2 The veal production chain: past and present figures

In Europe and North America, veal calves are usually surplus male and female calves in the dairy sector not needed for herd replacement that are raised up to 8 months of age for veal meat production (4). Veal is the pale-colored meat produced for centuries by fattening calves with solely cow’s milk. For these reasons, the veal production chain can be considered a sideline business of the dairy industry. In Europe, veal calves rearing has become important from the 1950s on, to handle the surplus of male calves of dairy breeds (mostly Holstein) and the excess of skimmed milk from the dairy industry. The introduction by the European Community of the milk quota system in 1984 severely affected the size of the dairy cattle population leading to a progressive decline of the veal industry around the year 1990 (5). Nowadays we can roughly estimate that around 4 million calves are raised in the European Union for veal production, with France, Netherlands, and Italy as the main producing Countries (6). Veal calf rearing is carried out according to very standardized procedures in specialized fattening units (7). In compliance with the Council Regulation EC/01/2005 on the protection of animals during transport, young calves leave their native dairy farms when they are 15–20 days old to be transferred to the specialized fattening units. Once at destination, calves are fattened for about 6 months under rigorous guidelines regarding biosecurity, housing structures, and feeding plans.

## 3 Why solid feeds in the veal calf’s diet?

For many years, the traditional veal calves feeding system consisted exclusively of a liquid diet based on whole cow’s milk. This was later replaced by milk replacers formulated with skimmed milk powder or whey powder and supplemented with lipid sources. Calves received two daily liquid meals, provided either in individual buckets or in a shared trough throughout the fattening period. Milk and milk replacers contain limited amounts of iron. The controlled provision of this trace element was intended to induce a gradual iron deficiency at the muscle level, thereby producing the pale meat color desired at slaughter. This feeding strategy has been widely criticized due to its negative implications for animal welfare (8). An all-liquid diet impedes the physiological development of the forestomach and prevents calves from expressing natural oral behaviors such as chewing and rumination. In the absence of SF, calves exhibit increased abnormal oral activities such as sucking, licking, or biting inanimate objects, as well as tongue rolling and tongue playing, as attempts to satisfy their motivation to ruminate (9). Liquid-fed calves also increase self-grooming and hair ingestion, which can result in the formation of hairballs (trichobezoars) in the rumen. These accumulations impair digestion and further compromise welfare (10).

## 4 European Union regulations and the research about the solids for veal calves

In response to the growing pressure from both public opinion and several non-governmental organizations for animal protection and welfare, the European Union (11) issued a Directive (91/629/EC) laying down the minimum standards for the protection of calves. Regarding calves’ feeding the Directives stated that “*all calves must be provided with an appropriate diet adapted to their age, weight and behavioural and physiological needs, to promote good health and welfare. To this end, […]. a minimum daily ration of fibrous food must be provided for each calf over two weeks old, the quantity being increased from 50 g to 250 g/d for calves from 8 to 20 weeks old […]*.” In parallel, the scientific research deepened the knowledge about the type of SF to be fed to the calves. The multidisciplinary EU project “Chain Management of Veal Calf Welfare” (12) compared several types of SF, having ad libitum hay and milk replacer alone as positive and negative controls, respectively. All the tested SF were provided in amounts of 250 and 500 g/calf/day but none of them fulfilled all the requirements of an “ideal” solid feed for veal calves (Table 1). However, the administration of SF had no detrimental effects on calves’ growth performance and promoted forestomach development. Moreover, SF promoted a marked reduction in the number of calves showing hairballs in the rumen, and this result was related to a continuous removal of ingested hair induced by the increased ruminal motility (12). Regarding veal meat quality, it has been demonstrated that there is not a straight-forward relationship between the iron content of the SF and the meat color, as especially in solids rich in structural carbohydrates (e.g., straw), iron is partially bound by the cell wall constituents, being less available for absorption and metabolism (13).

**Table 1:** Positive (+) or negative (−) effects on animal welfare traits and meat quality of the provision of different types of solid feeds to veal calves.

Type of roughage	Welfare traits			Production of pale-coloured meat
	Prevention of abnormal oral behavior	Promotion of rumen mucosal development	Prevention of the increase of abomasal lesions	
Hay *ad libitum*^1^	++	++	+	−
Straw: chopped	+	−	−	+
Straw: pellets	+/−	−	−	+
Dried maize silage	−	+	−	*
Dried maize cob silage	−	+	−	*
Fresh maize silage	+	+	−	*
Rolled barley	−	+/−	−	+
Dried beet pulps	−	−	−	−
Milk replacer alone^2^	−	−	+	+

1 Positive control.

2 Negative control.

*Meat colour strongly related to the Fe content of the roughage and the total Fe intake.

Summary of five multifactorial experiments carried out within the EU project “Chain management of veal calf welfare” modified from Blokhuis (12).

## 5 Farmers’ opinion about solid feeds: from an announced tragedy to an opportunity

Veal producers were extremely reluctant about the inclusion of SF in calves’ diets. In their opinion, the increased amount of iron brought by the solids would have led to carcasses and meat with an unacceptable red color, that would have been severely depreciated by the market. Further concerns regarded the potential interference of solids on milk consumption. According to farmers’ perceptions, the intake of solids would increase the frequency of ruminal drinking episodes, due to the impairment of oesophageal groove closure during milk intake. Ruminal drinking or milk leakage is a dysfunction causing the accumulation of milk in the forestomach (14). Its symptoms include inappétence, recurrent tympany, abdominal distension, growth retardation, clay-like faeces (15), and the most severe cases ruminal bloat (16). The entry into force across the EU countries of the Directives (11) for calf protection made mandatory the provision of SF, and what should have been a tragedy turned out to be an opportunity. Supported by several scientific studies (13, 17, 18), which proved that calves’ health and performance were not impaired by this new feeding program, white veal producers became more confident in the use of solids. From year to year, the daily dose of SF provided during the fattening cycle progressively increased, far beyond the legal duty, requiring the creation of a dedicated manger in each fattening pen. Behind this growing confidence there was the motivation that solids might also support the growth of the animals and this brought veal producers to prioritize the use of solids with high energy content, like starchy concentrates (maize grain or cereal mixtures). These feeds appeared to better match the dietary restriction in iron supply needed to produce pale-colored veal, while increasing rumen volatile fatty acids concentrations for faster body weight gain (18, 19). In the year 2011 (20), a comprehensive cross-sectional study monitored 170 veal farms in the main veal meat-producing countries in Europe (99 farms in the Netherlands, 47 in France, and 24 in Italy). The total amount of SF delivered to the calves during the whole fattening cycle was recorded and estimates about the average daily dose are reported in Table 2. More than 80% of the fattening units delivered a daily dose of solids far above the recommended amount of 250 g/calf set by EU Directive (11).

**Table 2:** Number and percentage of veal farms according to the total amount of dry matter (DM) from solid feeds administered to the veal calves throughout the fattening cycle and estimated daily dose of solids feed per calf modified from Brscic et al. (20).

Item	Total amount of solid feed (dry matter/calf)			
	≤50 kg	51–100 kg	101–150 kg	151–300 kg
Farms, *n* (%)	29 (17%)	39 (23%)	82 (48%)	20 (12%)
Mean daily dose^1^, g/calf/d	220	450	700	1,250

1 Estimate obtained by dividing the mean value by a standard number of 180 days of fattening.

## 6 New feeding plans and emerging welfare issues

The provision of large amounts of concentrate feed supported rumen development but simultaneously introduced new health and welfare challenges. It increased the risk of gastrointestinal disorders affecting both the rumen and the abomasum (20–22). Pre-ruminant calves have a limited rumen buffering capacity compared with older cattle. When exposed to high starch loads without an adequate intake of structured fiber, they are more prone to rapid drops in rumen pH due to shifts in fermentation and elevated lactic acid production (23). This acidic environment promotes hyperkeratinization of the rumen papillae, a process further exacerbated by the absence of the abrasive stimulation normally provided by roughage (24). The intake of large amounts of cereal-rich concentrates also predisposed calves to rumen plaque formation. These are areas of mucosa where coalescing papillae are covered by a sticky feed material, hair, and cellular debris (18, 20). Hyperkeratosis and plaques are among the earliest and most prominent macroscopic lesions of the rumen epithelium (25). A recent study (26) evaluated the effects of feeding veal calves large quantities of cereal-based solid feed on the prevalence of gastrointestinal lesions using post-mortem inspections at the abattoir. Trained veterinarians examined more than 600 rumens and abomasa from 41 batches originating from 28 veal farms. Descriptive information on the fattening cycle is presented in Table 3. Solid feed intake during the cycle was high and predominantly starch-based, with maize grain as the main component. The high prevalence of ruminal hyperkeratosis, plaques, and mucosal redness confirmed the detrimental effects of this feeding strategy on rumen integrity (Table 3).

**Table 3:** Descriptive statistics about the fattening cycle of veal calves belonging to 41 batches of animals from 28 veal farms that were inspected post-mortem at the abattoir to assess rumen mucosa disorders and abomasal lesions modified from Magrin et al. (26).

Item	Mean	Standard Deviation	Minimum	Maximum
Days of fattening, *n*	183	±19	140	225
Total milk replacer, kg	311	±31	258	365
Total solid feed, kg	158	±44	90	240
Prevalent type of solid feed^1^				
Maize grain, %	85	±16	15	93
Straw, %	8	±10	4	20
Rumen mucosa disorders				
Hyperkeratosis, % of rumens	60.1	±22.6	0.0	93.3
Plaques, % of rumens	68.2	±21.6	20.0	100
Sign of redness, % of rumens	27.9	±23.8	0	80.0
Abomasal lesions, % of abomasa				
≥1 lesion in the pyloric region	92.0	±9.02	61.1	100

1 Percentage of total solids.

## 7 Solid feeds and abomasa damage: meta-regression analysis

**Figure 1:**
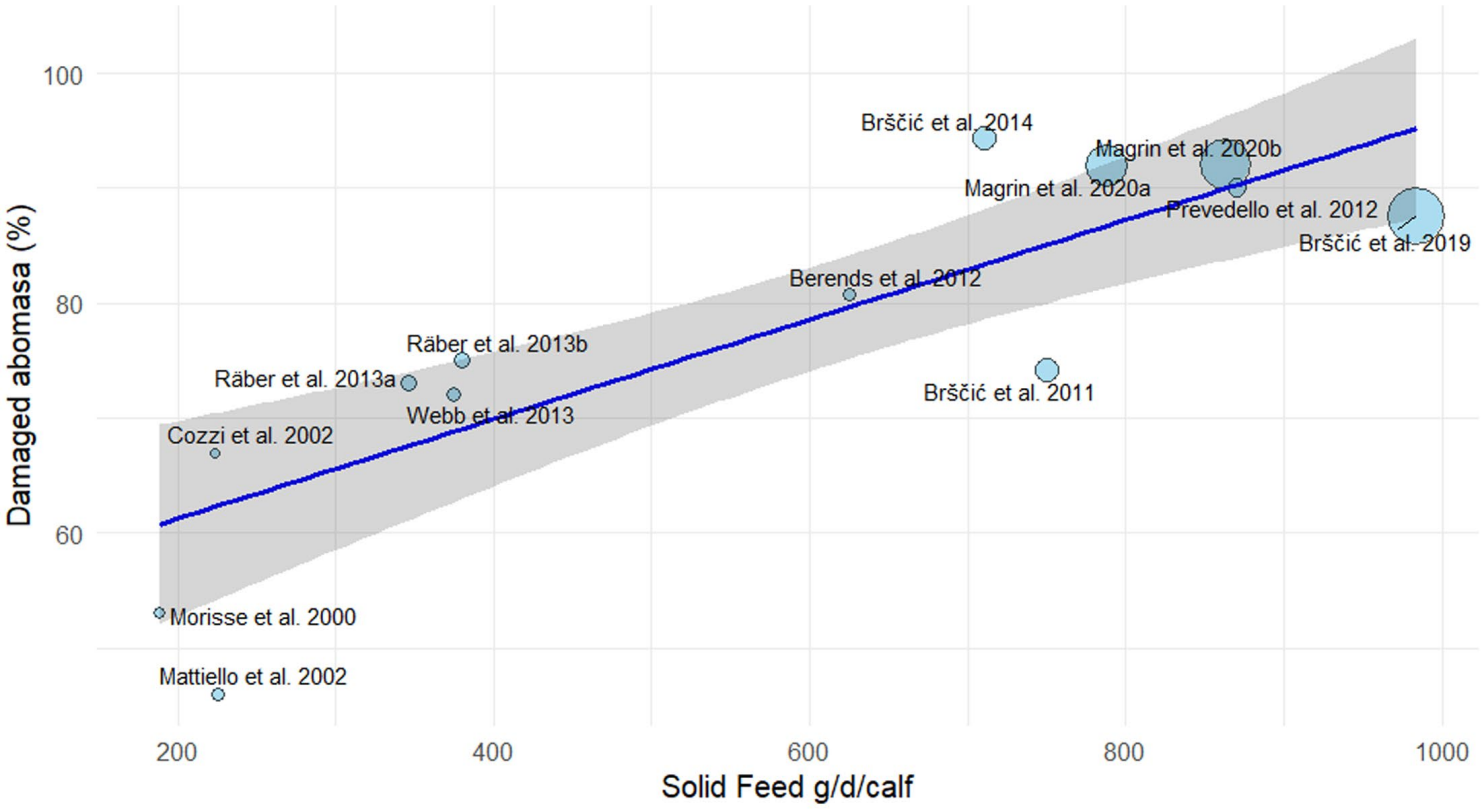
Linear meta-regression between the mean amount of solid feed administered to the veal calves during the fattening cycle and percentage of damaged abomasa. *Y* = 0.04X + 53; *R*^2^ = 0.71. The diameter of the bubbles in the cartesian plane is inversely proportional to variance of each study (bigger bubbles mean more precise study, i.e., smaller variance). The blue line shows the fitted meta-regression relationship. The shadowed area shows the 95% confidence interval.

In line with the outcomes of previous studies (27, 28), the provision of solid feed leads also to a high prevalence of abomasal lesions (Table 3). A previous review (22) has hypothesized that solid feeds might amplify abomasal damage that has already been caused by the ingestion of large quantities of milk replacer in two ways. The former is by causing a direct trauma, often referred in the literature as abrasion, to the abomasa wall. The latter is by blocking the pylorus, thereby delaying digesta from leaving the abomasum and exacerbating abomasal overloading by extending the time during which large quantities remain in the abomasum. To deepen the knowledge about the relation between amount and type of solid feed and abomasal damage in veal calves, we carried out a review of the existing literature. The first aim was to estimate the relationship between the amount of administered solid feed and abomasal damage. A further step aimed at investigating the relationship between abomasa damage and the amounts of single chemical components of the ingested solid: starch and fiber (NDF), in particular. To retrieve the data, the same search string (22) was applied to the Scopus database (Elsevier; accessed on 1 September 2025): [Abomas* AND (*damage* OR ulcer* OR lesion* OR scar*)] AND (*veal calf* OR *veal calves*). The search covered the period 2000–2024, yielding 29 documents. Exclusion criteria were: non-English language (two documents excluded) and review articles (one document excluded). In addition, eight studies did not report solid-feed data, three studies were not relevant to the research topic, and two studies were methodological papers without available data. Thirteen research papers reported the association between supplied SF and abomasa damage (Table 4). The amount of SF was calculated from the reported values in terms of dry matter (DM), starch and NDF per calf per day. When this information was not reported in a given paper, they were estimated by using other data (e.g., total amount of solid; duration of the cycle, number of animals, amounts supplied expressed as as-fed etc.). When not reported in the original study, reference values of chemical composition for different feed ingredients (29) were used to estimate the dry matter content of the SF. Likewise, the amounts of NDF and starch from SF consumed by the calves during the fattening period were estimated using the same feed composition database (29). In the case of mixed feeds, chemical composition values were estimated by weighting the tabular mean values of the individual ingredients according to their inclusion rates. As the protocols to define and detect the different lesions in the abomasa also varied between studies, in order to standardize and make the results comparable, an attempt was made to identify the percentage of affected abomasa for each paper (expressed as the percentage of total abomasa damaged out of the total inspected organs). Moreover, some of the selected articles reported different values for the abomasa damages caused by various tested solids. In these cases, the mean value of the reported percentages was considered. This decision was made because none of the articles reported a significant effect due to the different types of solids tested on the percentage of damage to the abomasum. The relationship between the mean daily amount of administered SF and the percentage of damaged abomasa was analyzed by a meta-regression analysis (30). We explored potential sources of heterogeneity using random-effects meta-regression model. All analyses were conducted in R using the metafor package (31). The results were shown in Figure 1.

**Table 4:** Articles retrieved from the Scopus® database (search conducted on 1 September 2025).

Article reference number	Authors and year of the study	Calves				Days of fattening	Diet solid feed^1^	Average solid feed intake			Inspected abomasa	
		Breed	Sex	Total number	Receiving solid feed (*n*)			DM	Starch	NDF	Total	Damaged
								g/calf/d			*n*	% ± SD
(10)	Morisse et al. (2000)	Friesian	Males	126	72	119	P1	189	91	49	21	29 ± 45
							P2	189	62	82	17	78 ± 41
(34)	Mattiello et al. (2002)	Polish Friesian	Males	138	92	153	BP	225	2	101	46	41 ± 49
							WS	227	2	175	46	52 ± 50
(35)	Cozzi et al. (2002)	Polish Friesian	Males	24	24	147	BG	219	131	33	12	75 ± 43
							STR	228	2	177	12	58 ± 49
(20)	Brscic et al. (2011)	Polish Friesian	Males	148	148	192	Mixed	750	232	307	148	74 ± 13
(27)	Berends et al. (2012)	Holstein Friesian	Males	106	80	84	MSC	625	170	256	40	89 ± 47
							MS	625	146	300	40	73 ± 47
(36)	Prevedello et al. (2012)	Polish Friesian	Males	78	78	206	CG	864	605	92	25	92 ± 12
							CGS	864	486	219	24	92 ± 20
							CGSES	883	456	249	26	85 ± 11
(37)	Räber et al. (2013a)	Swiss dairy and beef	Females/males	400	400	150	Mixed	346	63	164	346	73 ± 19
(38)	Räber et al. (2013b)	Swiss dairy and beef	Females/males	270	270	150	Mixed	380	71	193	252	75 ± 18
(28)	Webb et al. (2013)	Holstein Friesian	Males	300	240	168	Straw	375	4	300	80	73 ± 44
							MS	375	116	157	80	65 ± 48
							MC	375	75	225	80	78 ± 41
(39)	Brscic et al. (2014)	Polish Friesian	Males	79	79	201	CGS-U	711	424	146	52	96 ± 8
							CGS-EP	711	401	148	27	92 ± 11
(40)	Brscic et al. (2019)	Italian Holstein	Females/males	342	342	206	A	1,111	680	165	340	91 ± 4
				108	108	211	B	857	480	168	74	84 ± 3
(41)	Magrin et al. (2020a)	Italian Holstein	Males	416	416	185	CG	788	472	159	416	92 ± 9
(26)	Magrin et al. (2020b)	Italian Holstein	Males	656	656	183	CGS	863	545	147	656	92 ± 9

1 P1, agglomerated ground barley; P2, agglomerated ground barley + straw. BP, beet pulp; WS, wheat straw; BG, Barley grain; STR, straw; Mixed, mixture of concentrate and fibrous sources; MSC, maize silage, straw, and concentrate mixture; MS, maize silage; CG, corn grain; CGS, corn grain and straw; CGSES, corn grain, wheat straw, extruded soybean; MC maize cob silage; CGS-U, corn grain, straw, urea; CGS-EP, corn grain, straw, extruded pea; A, cereal grains mix; B, cereal grain mix and corn silage.

Main experimental settings of selected studies. Solid feed provided with milk replacer diet, estimated average intake of solid feed dry matter (DM), starch and NDF, total number of inspected abomasa and percentage of affected abomasa (% ± SD).

The meta-regression analysis produced an *R*^2^ value of 0.71. In the context of meta-regression, *R*^2^ represents the proportion of between-study heterogeneity that is accounted for by the moderator, in this case SF. Thus, including SF in the model reduced the unexplained heterogeneity by 71% compared with the intercept-only model. This indicates that differences in SF explain a substantial share of the true variability in effect sizes across the included studies, leaving only 29% of the original heterogeneity unexplained. The estimation of the slope coefficient was significant (*b* = 0.04 ± 0.01; *p* < 0.001). The data clearly show the trend over time towards the increase in the amount of solid feed included in calf diets. In the last 20 years, the average daily amount of solid feed administered to calves has increased by approximately 700 g. Over the same period, the prevalence of abomasal damage has risen by about 28 percentage points. The estimated value of the regression coefficient indicates that for every 100 g of DM increase in SF administered to the animals, the percentage of damaged abomasum increases by 4 percentage units.

**Figure 2:**
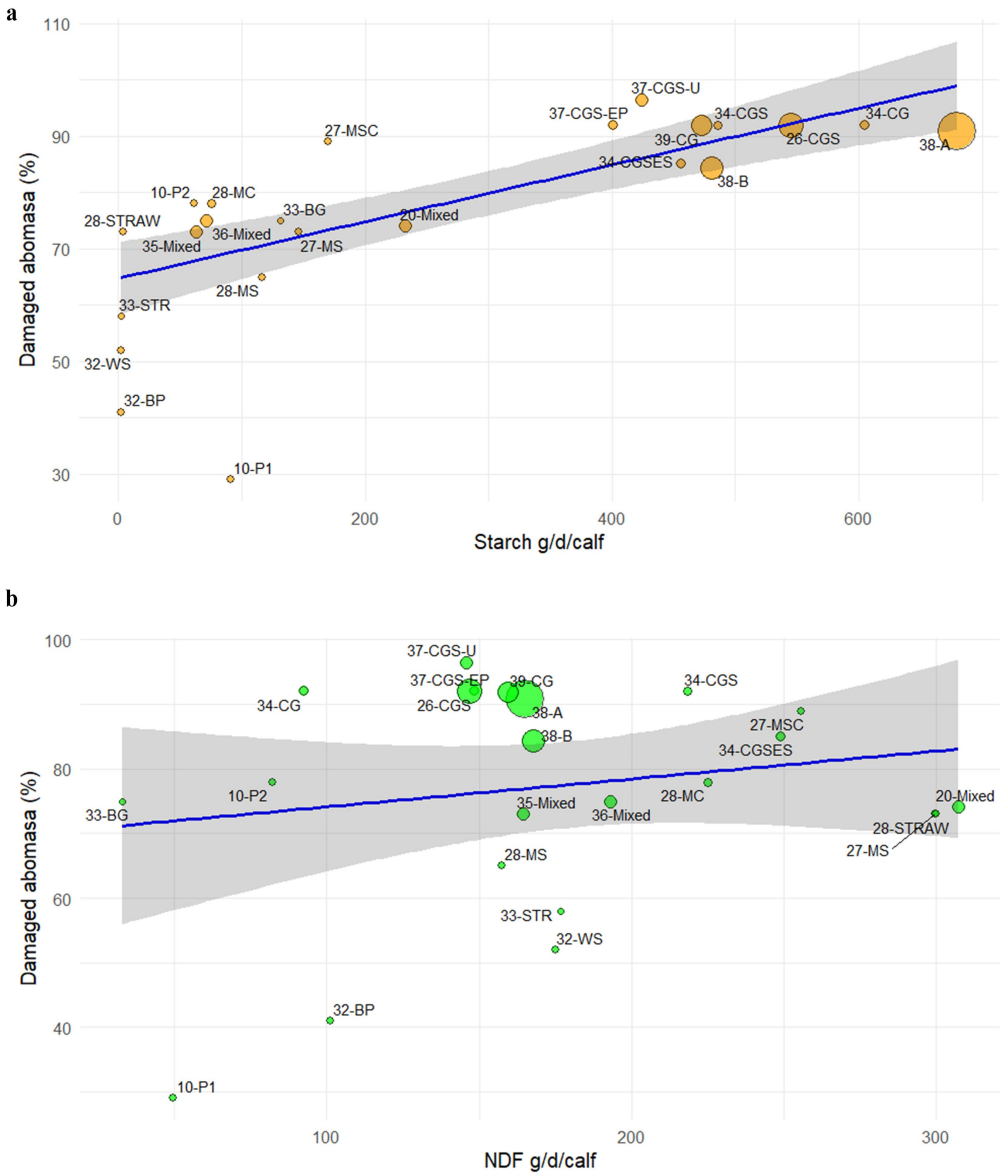
Linear meta-regression between the mean amount of **(a)** starch (*Y* = 0.05X + 65; *R*^2^ = 0.70) and **(b)** NDF (*Y* = 0.04X + 70; *R*^2^ = 0.01) administered to the veal calves through the solid feed during the fattening cycle and percentage of damaged abomasa. Bubbles are identified by reference number of the study – type of solid feed (Table 4). 10-P1: agglomerated ground barley; 10-P2: agglomerated ground barley + straw; 31-BP: beet pulp; 31-WS: wheat straw; 13-BG: Barley grain; 13-STR: straw; 20-/33-/34-Mixed: mixture of concentrate and fibrous sources; 28-STRAW: straw; 27-MSC: maize silage, straw, and concentrate mixture; 27-/28-MS: maize silage; 32-/37-CG: corn grain; 26-/32-CGS: corn grain and straw; 32-CGSES: corn grain, wheat straw, extruded soybean; 28-MC maize cob silage; 35-CGS-U: corn grain, straw, urea; 35-CGS-EP: corn grain, straw, extruded pea; 36-A: cereal grains mix; 36-B: cereal grain mix and corn silage.

The mean daily amounts of starch and NDF provided to the calves by SF in each study (Table 4) were associate with the percentage of affected abomasa using the meta-regression model. The relationship between starch and the percentage of damaged abomasa is significant (*p* < 0.001; Figure 2a), whereas the relationship between NDF and abomasal damage—is not (Figure 2b), supporting the hypothesis that it is primarily the amount of starch in the diet that determines the extent of abomasal damages.

The present review and meta-regression analysis provide new insights into the evolving role of solid feed in modern veal calf production and its implications for gastrointestinal health, with particular emphasis on abomasal integrity. Historically, the introduction of solid feed into veal production was motivated by welfare considerations, particularly the need to mitigate the negative behavioural and physiological consequences associated with milk-only diets (8, 9). However, quantitative and qualitative changes in solid feed administration have occurred over the past two decades with an increase in their amount and energy density. This trend was driven primarily by economic factors, particularly the frequent increases in the cost of skimmed milk powder and whey powder, main raw materials used in milk replacer formulations for calves. These price dynamics encouraged the partial substitution of milk replacer with SF (32). In addition, the shift was supported by scientific evidences, reporting improved performance in veal calves when receiving high levels of SF particularly at the end of the fattening period (27, 33). The synthesis of the available literature confirmed that providing SF promoted rumen development (13, 18) and reduced abnormal oral behaviors in veal calves (34). However, the increase amount of their daily intake shifted the welfare challenges for veal calves from behavioural deprivation towards gastrointestinal dysfunction. The meta-regression analysis carried out in this study has shown that this shift has promoted a marked increase in the prevalence of abomasal lesions recorded at slaughter. Moreover, the strong association identified between starch intake and abomasal damage suggests that under current solid feeding practices, the starch load delivered to the abomasum, rather than the physical abrasiveness of dietary fiber, appears the primary driver of abomasal lesions in veal calves. This significant association between increasing starch intake and lesion prevalence, coupled with the absence of a comparable relationship for NDF, indicates that starch-induced acidification is likely a central pathogenic mechanism.

The findings of this review should be interpreted within the broader framework of animal welfare. The introduction of SF into veal calf diets imposed by European Union welfare regulation was based on strong scientific evidence showing clear welfare benefits at low inclusion levels. Current issues are therefore not inherent to solid feeding itself, but they arise from the implementation of high-starch feeding plans. This distinction is essential for informing evidence-based policy and on-farm management. The rising prevalence of abomasal lesions should not prompt the abandonment of solid feeding as a welfare measure. Instead, it indicates the need to optimise solid feeding strategies. In line with this statement, the recent welfare opinion (6) emphasized the need to provide veal calves with long-fiber roughages, such as hay, as well as more frequent meals or to extend access to feed. Findings from the present study further suggest to lower the current dietary starch load due to its detrimental effects on the structural integrity of both the rumen wall and the abomasum.

## 8 Study limitations

Further research is needed to address several limitations inherent in the present study. The main limitation arises from the reduced number of studies that report, with adequate precision, the quantities of SF administered to calves and its detailed chemical composition, including particle size. Beyond the quantity, it is also essential to clearly figure out if the timing of SF administration, compared to milk feeding, may influence the development of abomasal damage. Regarding the assessment of abomasal lesions, the current lack of standardized protocols for their evaluation in veal calves significantly limits the ability to precisely clarify the relationship between SF administration and the type and severity of different pathologies.

## 9 Final remarks

This review of solid feeding of beef calves shows that the definition of welfare for a specific category of cattle is dynamic and can change with the adoption of new farming techniques. In fact, while feeding small amounts of SF improved calf welfare compared to milk-only diets, current feeding practices involving large amounts of high-starch SF have introduced new health risks, particularly with regard to the integrity of the rumen wall and the abomasum. The results of the meta-regression analysis indicated that starch intake, rather than NDF from SF, was significantly associated with the occurrence of abomasal damages. This suggests the need to re-evaluate both the composition and feeding protocols of SF to align the modern veal supply chain with the principles of sustainable and ethical farming.

## References

[1] Ten Napel J van der Veen AA Oosting SJ Koerkamp PWG. A conceptual approach to design livestock production systems for robustness to enhance sustainability. Livest Sci. (2011) 139:150–60. doi: 10.1016/j.livsci.2011.03.007

[2] Buller H Blokhuis H Jensen P Keeling L. Towards farm animal welfare and sustainability. Animals. (2018) 8:81. doi: 10.3390/ani8060081, 29799456 PMC6025272

[3] Nalon E Contiero B Gottardo F Cozzi G. The welfare of beef cattle in the scientific literature from 1990 to 2019: a text mining approach. Front Vet Sci. (2021) 7:588749. doi: 10.3389/fvets.2020.588749, 33505997 PMC7832582

[4] Webb LE Verwer C Bokkers EAM. The future of surplus dairy calves – an animal welfare perspective. Front Anim Sci. (2023) 4:1228770. doi: 10.3389/fanim.2023.1228770

[5] de Boer T. Veal production in the European Community In: Metz JHM Groenestein CM, editors. New trends in veal calf production. Wageningen: Wageningen Academic Publishers (1991). 8–15.

[6] European Food Safety Authority. Welfare of calves – scientific opinion of the AHAW panel on animal health and welfare. EFSA J. (2023) 21:7896. doi: 10.2903/j.efsa.2023.7896

[7] Cozzi G Brscic M Gottardo F. Main critical factors affecting the welfare of beef cattle and veal calves raised under intensive rearing systems in Italy: a review. Ital J Anim Sci. (2009) 8:67–80. doi: 10.4081/ijas.2009.s1.67

[8] Broom DM. Needs and welfare of housed calves In: Metz JHM Groenestein CM, editors. New trends in veal calf production. Wageningen: EAAP Publications (1991). 23–31.

[9] Sambraus HH. Mouth-based anomalous syndromes In: Fraser AF, editor. Ethology of farm animals: a comprehensive study of the behavioural features of common farm animals. World Animal Science A5 Amsterdam: Elsevier (1985). 391–422.

[10] Morisse JP Huonnic D Cotte JP Martrenchar A. The effect of four fibrous feed supplementations on different welfare traits in veal calves. Anim Feed Sci Technol. (2000) 84:129–36. doi: 10.1016/S0377-8401(00)00112-7, 40069496

[11] European Union. Council directive 91/629/EEC of 19 November 1991 laying down minimum standards for the protection of calves. Off J Eur Comm. (1991) 340:28–32.

[12] Blokhuis HJ. Chain Management of Veal Calf Welfare: Final report of the EU project FAIR3-PL96-2049. Lelystad: Animal Sciences Group, Wageningen UR (2000).

[13] Cozzi G Gottardo F Mattiello S Canali E Scanziani M Andrighetto I. The provision of solid feeds to veal calves: I. Growth performance, forestomach development, and carcass and meat quality. J Anim Sci. (2002) 80:357–66. doi: 10.2527/2002.802357x, 11881925

[14] Labussière E Berends H Gilbert MS van den Borne JJG Gerrits WJJ. Estimation of milk leakage into the rumen of milk-fed calves through an indirect and repeatable method. Animal. (2014) 8:1643–52. doi: 10.1017/S1751731114001670, 25231281

[15] van Weeren-Keverling Buisman A Wensing T Breukink HJ Mouwen JMVM. Ruminal drinking in veal calves In: Metz JHM Groenestein CM, editors. New trends in veal calf production. Wageningen: EAAP Publications (1991). 113–7.

[16] Kaba T Abera B Kassa T. Esophageal groove dysfunction: a cause of ruminal bloat in newborn calves. BMC Vet Res. (2018) 14:276. doi: 10.1186/s12917-018-1573-2, 30200937 PMC6131847

[17] Di Giancamillo A Bosi G Arrighi S Savoini G Domeneghini C. The influence of different fibrous supplements in the diet on ruminal histology and histometry in veal calves. Histol Histopathol. (2003) 18:727–33. doi: 10.14670/HH-18.727, 12792884

[18] Suárez BJ Van Reenen CG Stockhofe N Dijkstra J Gerrits WJ. Effect of roughage source and roughage to concentrate ratio on animal performance and rumen development in veal calves. J Dairy Sci. (2006a) 90:2390–403. doi: 10.3168/jds.2006-524, 17430943

[19] Suárez BJ Van Reenen CG Gerrits WJJ Stockhofe N van Vuuren AM Dijkstra J. Effects of supplementing concentrates differing in carbohydrate composition in veal calf diets: II. Rumen development. J Dairy Sci. (2006b) 89:4376–86. doi: 10.3168/jds.S0022-0302(06)72484-5, 17033025

[20] Brscic M Heutinck LFM Wolthuis-Fillerup M Stockhofe N Engel B Visser EK . Prevalence of gastrointestinal disorders recorded at postmortem inspection in white veal calves and associated risk factors. J Dairy Sci. (2011) 94:853–63. doi: 10.3168/jds.2010-3480, 21257054

[21] Berends H Van den Borne JJGC Mollenhorst H Van Reenen CG Bokkers EAM Gerrits WJJ. Utilization of roughages and concentrates relative to that of milk replacer increases strongly with age in veal calves. J Dairy Sci. (2014) 97:6475–84. doi: 10.3168/jds.2014-8098, 25129492

[22] Bus JD Stockhofe N Webb LE. Invited review: Abomasal damage in veal calves. J Dairy Sci. (2018) 102:943–60. doi: 10.3168/jds.2018-15292, 30591333

[23] Bertram HC Kristensen NB Vestergaard M Jensen SK Sehested J Nielsen NC . Metabolic characterization of rumen epithelial tissue from dairy calves fed different starter diets using ^1^H NMR spectroscopy. Livest Sci. (2009) 120:127–34. doi: 10.1016/j.livsci.2008.05.001

[24] Beharka AA Nagaraja TG Morrill JL Kennedy GA Klemm RD. Effects of form of the diet on anatomical, microbial, and fermentative development of the rumen of neonatal calves. J Dairy Sci. (1998) 81:1946–55. doi: 10.3168/jds.S0022-0302(98)75768-6 9710764

[25] Vestergaard M Jarltoft TC Kristensen NB Børsting CF. Effects of rumen-escape starch and coarseness of ingredients in pelleted concentrates on performance and rumen wall characteristics of rosé veal calves. Animal. (2013) 7:1298–306. doi: 10.1017/S1751731113000414, 23506959

[26] Magrin L Brscic M Cozzi G Armato L Gottardo F. Prevalence of gastrointestinal, liver and claw disorders in veal calves fed large amounts of solid feed through a cross-sectional study. Res Vet Sci. (2020b) 133:318–25. doi: 10.1016/j.rvsc.2020.10.022, 33153761

[27] Berends H Van Reenen CG Stockhofe-Zurwieden N Gerrits WJJ. Effects of early rumen development and solid feed composition on growth performance and abomasal health in veal calves. J Dairy Sci. (2012) 95:3190–9. doi: 10.3168/jds.2011-4643, 22612954

[28] Webb LE Bokkers EAM Heutinck LFM Engel B Buist WG Rodenburg TB . Effects of roughage source, amount, and particle size on behavior and gastrointestinal health of veal calves. J Dairy Sci. (2013) 96:7765–76. dx.doi: 10.3168/jds.2012-6135. doi: 10.3168/jds.2012-6135, 24094537

[29] National Academies of Sciences, Engineering, and Medicine. Nutrient requirements of dairy cattle: Eighth revised edition. Washington, DC: The National Academies Press (2021). 38386771

[30] Thompson SG Higgins JPT. How should meta-regression analyses be undertaken and interpreted? Stat Med. (2002) 21:1559–73. doi: 10.1002/sim.1187, 12111920

[31] Viechtbauer W. Conducting meta-analyses in R with the metafor package. J Stat Softw. (2010) 36:1–48. doi: 10.18637/jss.v036.i03

[32] Mollenhorst H Berentsen PBM Berends H Gerrits WJJ de Boer IJM. Economic and environmental effects of providing increased amounts of solid feed to veal calves. J Dairy Sci. (2016) 99:2180–9. doi: 10.3168/jds.2014-9212, 26805966

[33] Xie XX Meng QX Liu P Wu H Li SR Ren LP . Effects of a mixture of steam-flaked corn and extruded soybeans on performance, ruminal development, ruminal fermentation, and intestinal absorptive capability in veal calves. J Anim Sci. (2013) 91:4315–21. doi: 10.2527/jas.2012-5731, 23881685

[34] Mattiello S Canali E Ferrante V Caniatti M Gottardo F Cozzi G . The provision of solid feeds to veal calves: II. Behavior, physiology, and abomasal damage. J Anim Sci. (2002) 80:367–75. doi: 10.2527/2002.802367x, 11881926

[35] Cozzi G Gottardo F Mutinelli F Contiero B Fregolent G Segato S . Growth performance, behaviour, forestomach development and meat quality of veal calves provided with barley grain or ground wheat straw for welfare purpose. Ital J Anim Sci. (2002) 1:113–26. doi: 10.4081/ijas.2002.113

[36] Prevedello P Brscic M Schiavon E Cozzi G Gottardo F. Effects of the provision of large amounts of solid feeds to veal calves on growth and slaughter performance and intravitam and postmortem welfare indicators. J Anim Sci. (2012) 90:3538–46. doi: 10.2527/jas.2011-4666, 22585794

[37] Räber R Kaufmann T Regula G von Rotz A Stoffel MH Posthaus H . Effects of different types of solid feeds on health status and performance of Swiss veal calves. I. Basic feeding with milk by-products. Schweiz Arch Tierheilkd. (2013a) 155:269–81. doi: 10.1024/0036-7281/a000458 23644290

[38] Räber R Kaufmann T Regula G von Rotz A Stoffel MH Posthaus H . Effects of different types of solid feeds on health status and performance of swiss veal calves. II. Basic feeding with whole milk. Schweiz Arch Tierheilkd. (2013b) 155:283–92. doi: 10.1024/0036-7281/a000459 23644291

[39] Brscic M Prevedello P Stefani A Cozzi G Gottardo F. Effects of the provision of solid feeds enriched with protein or nonprotein nitrogen on veal calf growth, welfare, and slaughter performance. J Dairy Sci. (2014) 97:4649–57. doi: 10.3168/jds.2013-7618, 24819140

[40] Brscic M Magrin L Prevedello P Pezzuolo A Gottardo F Sartori L . Effect of the number of daily distributions of solid feed on veal calves’ health status, behaviour, and alterations of rumen and abomasa. Ital J Anim Sci. (2019) 18:226–35. doi: 10.1080/1828051X.2018.1504634

[41] Magrin L Gottardo F Contiero B Cozzi G. Association between gastrointestinal tract, claw disorders, on-farm mortality and feeding management in veal calves. Ital J Anim Sci. (2020a) 20:6–13. doi: 10.1080/1828051X.2020.1863868, 41307611

